# Impfbereitschaft von Eltern mit einem COVID-19-Vakzin: Die Rolle von Elternschaft und Geschlecht

**DOI:** 10.1007/s00608-021-00925-2

**Published:** 2021-10-01

**Authors:** Amelie Altenbuchner, Sonja Haug, Rainer Schnell, Anna Scharf, Karsten Weber

**Affiliations:** 1 Institut für Sozialforschung und Technikfolgenabschätzung (IST), Regensburg Center of Health Sciences and Technology (RCHST), Ostbayerische Technische Hochschule (OTH) Regensburg, Seybothstr. 2, 93953 Regensburg, Deutschland; 2 Research Methodology Group, Universität Duisburg-Essen, Forsthausweg 2, 47057 Duisburg, Deutschland

**Keywords:** Nebenwirkungen, Impfstoffverschwörungsglaube, Einstellung zu Homöopathie, Risikokommunikation, Impfberatung, Side effects, Vaccine conspiracy beliefs, Attitude towards homeopathy, Risk communication, Vaccination advice

## Abstract

Hintergrund. Eltern stehen im Rahmen der eigenen Impfung und der Kinderimpfung mit einem COVID-19-Vakzin vor einer Impfentscheidung. Zum aktuellen Zeitpunkt gibt es keine (vollständige) Impfempfehlung.

Fragestellung. Die Studie untersucht die Impfbereitschaft von Eltern minderjähriger Kinder und Personen ohne minderjährige Kinder, wobei insbesondere Geschlechtsunterschiede überprüft werden.

Methoden. Die Studie basiert auf einer Zufallsstichprobe (Telefon-Survey, *n* = 2014, Erhebung zwischen 12.11.2020 und 10.12.2020). Die Auswertung stützt sich insbesondere auf die Teilstichprobe von Personen mit minderjährigen Kindern im Haushalt (*n* = 461).

Ergebnisse. Eltern weisen durchgängig eine geringere Impfbereitschaft mit einem COVID-19-Vakzin auf als Befragte ohne minderjährige Kinder (54,1 % vs. 71,1 %). Väter weisen eine stärker ausgeprägte eigene Impfbereitschaft auf als Mütter. Darüber hinaus sind Männer eher als Frauen bereit, das eigene Kind mit einem COVID-19-Vakzin impfen zu lassen.

Schlussfolgerungen. Bei Eltern und insbesondere Müttern ist eine erhebliche Fehleinschätzung von Impfrisiken und häufiger Glaube an Impfverschwörungstheorien zu beobachten. Empfohlen werden anschauliche und leicht verständliche Informationen über die Wirkung und Nebenwirkungen der Impfung mit einem COVID-19-Vakzin durch zuständige Institutionen und Ärzte.

## Abstract (en)

Background. Parents are faced with a vaccination decision in the context of their own vaccination and that of their children with a COVID-19 vaccine. At present, there is no (complete) vaccination recommendation.

Research question. The study investigates the willingness to vaccinate of parents of minors and people without children who are minors, in which gender differences in particular are examined.

Methods. The study is based on a random sample (telephone survey, *n* = 2,014, collected between 12 November and 10 December 2020). The evaluation is primarily based on the sub-sample of people with minors in the household (*n* = 461).

Results. Parents of minors consistently show a lower willingness to be vaccinated with a COVID-19 vaccine than respondents without minors (54.1% vs 71.1%). Fathers show a stronger willingness to be vaccinated than mothers. Furthermore, men are more willing to get their own child vaccinated with a COVID-19 vaccine than are women.

Conclusions. Among parents and especially mothers, a considerable misrepresentation of vaccination risks and frequent beliefs in vaccination conspiracy theories can be observed. Clear and easily understandable information on the effects and side effects of vaccination with a COVID-19 vaccine by relevant institutions and physicians is recommended.

Die Impfentscheidung für minderjährige Kinder liegt bei den Eltern. Hierbei zeigt sich insbesondere das empfundene Vertrauen in Impfstoffe und das Gesundheitssystem als bedeutsamster Einflussfaktor. Die Nebenwirkungen werden überschätzt und individuelle Erkrankungsrisiken durch Impflinge bzw. Eltern unterschätzt [11]. Am 28.05.2021 genehmigte die Europäische Arzneimittelagentur (EMA) die Zulassung des COVID-19-Impfstoffs Comirnaty von Biontech/Pfizer für Kinder ab 12 Jahren [5]. Die Ständige Impfkommission (STIKO) in Deutschland [20] empfiehlt die Impfung für gesunde Kinder nicht (Stand: 10.06.2021), jedoch das Nationale Impfgremium ([3]; Stand: 24.06.2021). Somit gilt für Ärztinnen und Ärzte, individuelle informierte Entscheidungen der Eltern zu fördern, obgleich die Evidenzlage in der besonderen Situation der COVID-19-Pandemie unvollständig ist [13].

## Methode

Die Studie basiert auf einer Ein-Themen-Bevölkerungsbefragung zur Impfbereitschaft (*n* = 2014; [9]). Der deutschlandweite Telefon-Survey auf Basis einer Zufallsstichprobe („dual frame“) fand zwischen dem 12.11.2020 und 10.12.2020 statt. Die Altersspanne umfasst 18–95 Jahre (Medianalter 52). Von den Befragten sind 50,8 % weiblich, 49 % männlich und 0,1 % divers. Die vorliegende Teilstichprobe von Personen mit minderjährigen Kindern im Haushalt (*n* = 461; 22,9 %; folgend Eltern) unterscheidet sich in Bezug auf den Frauen- (53,8 %) und Männeranteil (46,2 %) nicht signifikant von der Vergleichsgruppe ohne minderjährige Kinder. Das Durchschnittsalter der Eltern ist 40,1 Jahre (*SD* = 11,4), der Kinderlosen 54,2 Jahre (*SD* = 19,6).

## Ergebnisse

Eine Analyse der oben genannten Bevölkerungsbefragung ergab, dass die Impfbereitschaft mit einem COVID-19-Vakzin mit dem Alter steigt, eine erhöhte und unrealistische Wahrnehmung schwerwiegender Nebenwirkungen von Impfungen mit sinkender Impfbereitschaft einhergeht, bei der individuellen Risikoeinschätzung einer Erkrankung mit COVID-19 Unsicherheiten bestehen, da über die Hälfte der Befragten die langfristigen Konsequenzen einer COVID-19-Erkrankung nur schwer einschätzen kann, und Personen mit hohen Zustimmungswerten zu (impf‑)verschwörungstheoretischen Aussagen eine niedrige Impfbereitschaft zeigen. Ein Viertel der Impfablehnenden sagt, dass sie nicht glauben, dass das Coronavirus gefährlich für sie sei [9]. Am häufigsten nennen sie, ebenso wie Befragte anderer Studien [16], die Sorge vor Nebenwirkungen.

### Impfbereitschaft der Eltern

Die Impfbereitschaft der Befragten mit und ohne Kinder zeigt einen signifikanten Unterschied (53,9 % vs. 71,1 % ja sicher/eher ja). Die Impfbereitschaft der Eltern ist auch nach einer multiplen logistischen Regressionsanalyse signifikant niedriger als die der Befragten ohne Kinder (Kovariaten: Alter, Geschlecht, Schulabschluss, Risikogruppenzugehörigkeit, wahrgenommenes Risiko, Konsequenzen einer Erkrankung, eigene COVID-19-Infektion, Infektion einer Person im sozialen Umfeld, wahrgenommene Wahrscheinlichkeit ernster Nebenwirkungen bei Grippeimpfungen, Vertrauen in das Robert Koch-Institut [RKI], Impfbereitschaft sozialer Bezugsgruppen).

In der Subpopulation der Eltern ist – wie in der Gesamtstichprobe – ein signifikanter Geschlechtsunterschied zu beobachten. Die Hälfte der Frauen und 58,4 % der Männer sind sicher oder eher impfbereit. Hierbei sind Frauen zu einem geringeren Teil sicher zur Impfung bereit (20,5 %) als Männer (33,0 %). Das Alter von Eltern hängt nicht mit ihrer Impfbereitschaft zusammen. Die Impfbereitschaft der Eltern steigt mit dem Schulabschluss mit Ausnahme der Fachhochschulreife (Impfbereitschaft bei Abitur: 61,6 %, bei Fachhochschulreife: 41,8 %, bei mittlerer Reife: 49,4 %, bei Hauptschulabschluss: 45,9 %). Die Impfbereitschaft ist bei Befragten mit Hochschulabschluss (70,9 %) signifikant höher als bei Befragten ohne Hochschulabschluss (48,5 %).

Impfablehnende Eltern, die sich eher oder sicher nicht impfen lassen wollen (*n* = 204), nennen am häufigsten die Angst vor Nebenwirkungen (34,6 %) und den Glauben an die Ungefährlichkeit des Coronavirus für sie (12,8 %). Männer (37,9 %) halten das Coronavirus häufiger für ungefährlich als Frauen (22,2 %). Frauen (88,0 %) nennen signifikant häufiger als Männer Nebenwirkungen als Grund (64,4 %). Dennoch würden sich 23,6 % impfen lassen, wenn das Nebenwirkungsrisiko nicht größer als bei einer Grippeschutzimpfung wäre, und 19,7 %, wenn die Impfung zuverlässig vor einer Coronainfektion schützt. Die Impfschutzzuverlässigkeit ist für Frauen (51,5 %) signifikant bedeutsamer als für Männer (26,6 %).

### Impfung der Kinder mit einem COVID-19-Vakzin

Die eigene Impfbereitschaft steht in einem positiven Zusammenhang mit der Bereitschaft, die Kinder impfen zu lassen: 52,3 % würden ihre Kinder sicher oder eher mit einem COVID-19-Vakzin impfen lassen, 29,3 % eher nicht und 18,3 % sicher nicht. Hierbei zeigt sich ein signifikanter Geschlechtseffekt: 39,4 % der Männer mit Kindern würden ihre Kinder sicher impfen lassen (eher ja 18,8 %). Bei Frauen sind insgesamt 47,6 % impfbereit (ja sicher 21 %, eher ja 26,6 %). Ihre Kinder würden 22,3 % der Frauen sicher nicht impfen lassen (30,1 % eher nicht). Dies gilt nur für 13,4 % der Männer (28,4 % eher nicht).

### Einschätzung von Nebenwirkungen und Impfkomplikationen

Zum Zeitpunkt der Befragung war noch kein COVID-19-Vakzin in der EU zugelassen. Es wurde nach der Einschätzung der Häufigkeit ernster Nebenwirkungen bei Grippeimpfungen gefragt, da hierzu Erfahrungen in der Bevölkerung vorliegen [4, 17]. In der Gesamtstichprobe zeigt sich eine vollkommen überschätzte Wahrnehmung der Wahrscheinlichkeit ernster Nebenwirkungen (Median 20 %; [9]) (vgl. auch [12] – ohne signifikante Geschlechtsunterschiede sowie zwischen Eltern und anderen Befragten. Jedoch tritt bei Eltern ein signifikanter Geschlechtsunterschied auf: Mütter vermuten ernsthafte Nebenwirkungen durchschnittlich bei 30,9 % der Fälle (*SD* = 24,6), Väter bei 24,4 % (*SD* = 24,7). Es liegt ein Interaktionseffekt zwischen Geschlecht und Hochschulabschluss vor (Mütter mit Hochschulabschluss: 29,8 %; Väter mit Hochschulabschluss: 8,5 %). Auch höher gebildete Frauen sind somit signifikant schlechter informiert als höher gebildete Männer.

In der Gesamtstichprobe korreliert die Zustimmung zur Vaccine Conspiracy Beliefs Scale (VCBS; [23]) und die Überschätzung der Wahrscheinlichkeit von Impfnebenwirkung. Eltern und insbesondere Mütter stimmen den Items der VCBS häufig zu (Tab. 1).

**Table 1:** 

	Stimme überhaupt nicht zu	Stimme eher nicht zu	Stimme eher zu	Stimme voll zu	Gesamt
Pharmaunternehmen spielen die Gefahren von Impfstoffen herunter (*n* = 421)	11,6	31,4	34,4	22,6	100,0
Nebenwirkungen von Impfungen werden häufig verschwiegen (*n* = 441)	20,6	27,9	25,6	25,9	100,0
Die Wirksamkeit von Impfstoffen wird häufig übertrieben (*n* = 450)*; Zustimmung Frauen > Männer	21,3	43,8	21,2	13,7	100,0
Es wird versucht, einen Zusammenhang zwischen Impfstoffen und Autismus zu vertuschen (*n* = 322)*; Zustimmung Frauen > Männer	45,8	34,7	11,4	8,1	100,0

Items nach [23], *n* Fallzahl. Geschlechtsunterschied (Kruskall-Wallis *H*) ***p < 0,001, **p < 0,01, *p < 0,05

### Besuche bei Allgemein- und Facharzt sowie Haltung zu Homöopathie

In der Gesamtstichprobe erhöht sich mit steigender Häufigkeit von Arztbesuchen die Wahrscheinlichkeit zur Gruppe der Impfbereiten zu gehören. Die befragten Mütter suchten in den letzten 12 Monaten im Durchschnitt 4,7-mal (*SD* = 6,9) und somit signifikant häufiger einen Allgemein- oder Facharzt auf als Väter (*M* = 3,2, *SD* = 3,7). Jedoch erhöhen häufige Arztbesuche in der Subgruppe der Eltern deren Impfbereitschaft sowie die Bereitschaft zur Kinderimpfung nicht signifikant.

In der Gesamtstichprobe ist mit Ablehnung „alternativer Heilmethoden“ eine signifikant höhere Impfbereitschaft verbunden. Ebenfalls besteht ein signifikanter Zusammenhang zwischen der Haltung zur Homöopathie und der eigenen Impfbereitschaft: Wird Homöopathie befürwortet, ist die Impfbereitschaft geringer. Auch in der Teilstichprobe der Eltern zeigt sich dieser Zusammenhang zwischen der Haltung zu Homöopathie und Impfbereitschaft (Tab. 2). Unter Eltern sind es wiederum Frauen, die signifikant häufiger eine positive Haltung zur Homöopathie haben als Männer, die häufiger gar nichts davon halten.

**Table 2:** 

Wenn ein Impfstoff gegen das Coronavirus in Deutschland zugelassen wird: Würden Sie sich impfen lassen?	Was halten Sie von Homöopathie?		
	Viel	Etwas	Gar nichts
Sicher nicht	40,9	15,0	10,0
Eher nein	21,5	29,6	26,0
Eher ja	17,2	40,8	20,0
Ja sicher	20,4	14,6	44,0
Impfbereitschaft (ja sicher, eher ja)	37,6	55,4	64,0
Impfablehnung (eher nicht, sicher nicht)	62,4	44,6	36,0
Gesamt	100,0	100,0	100,0

*n* = 375; Kruskall-Wallis-Test *p* < 0,001

## Diskussion

Die Impfbereitschaft von Eltern war zum Zeitpunkt der Befragung insgesamt geringer als in der Vergleichsgruppe. Hierbei zeigten Väter eine höhere Impfbereitschaft als Mütter. Das gilt auch für die Bereitschaft, die eigenen Kinder impfen zu lassen. Der Befund ist konsistent mit der geringeren Impfbereitschaft von Frauen in einer internationalen Metaanalyse zu COVID-19-Befragungen [21].

Insgesamt zeigen sich die Befragten bei der Impfentscheidung als rationale Akteurinnen und Akteure. Personen aus Risikogruppen und solche, die die Gefährlichkeit einer COVID-19-Erkrankung hoch einschätzen, haben eine hohe Impfbereitschaft [9]. Dem steht entgegen, dass in der Bevölkerung die Wahrscheinlichkeit von Nebenwirkungen und das Impfrisiko überschätzt werden [9, 11, 13, 15]. Von Eltern wird die Wahrscheinlichkeit ernster Nebenwirkungen bei Grippeimpfungen sehr unrealistisch und viel zu hoch eingeschätzt, was bei Müttern am stärksten ausgeprägt ist. Mütter besuchen häufiger Ärztinnen und Ärzte und haben insgesamt eine positivere Einstellung zur Homöopathie. Ein gewisses Vertrauen in nicht evidenzbasierte Methoden wie der Homöopathie [7] zeigt sich insbesondere bei höher gebildeten Eltern mit höherem sozialen Status [15] (vgl. auch [18]). Der Ursprung für den Wunsch nach homöopathischer Behandlung der Kinder liegt häufig in der Vermeidung von Nebenwirkungen [1, 7].

Schlussendlich sind es Ärzte, die durch ihre Rolle als bedeutsamste Vertrauensperson die Impfbereitschaft der Eltern unmittelbar beeinflussen können [10, 11]. Für eine informierte Entscheidung sind vollständige und transparente Informationen sowie ein Verständnis von Gesundheitsstatistiken erforderlich [6, 9]. Eine realistische Nutzen- und Risikoeinschätzung könnte in der Beratung und Aufklärung der Eltern (z. B. durch „Icon Arrays“; [14]) partnerschaftlich besprochen werden, um Sorgen und Ängsten entgegenzuwirken [8] und, kindgerecht aufbereitet, zur Aufklärung der betreffenden Kinder bzw. Jugendlichen [22] beizutragen.

Forschungsbedarf ergibt sich in Bezug auf die Ursachen der Einstellungsmuster der Eltern, beispielsweise den Einfluss der Einstellung von Hebammen zu Impfungen [24]. Aktuell besteht bei Schwangeren und Stillenden, denen aufgrund eines erhöhten Ansteckungs- oder Krankheitsverlaufsrisikos im Bedarfsfall eine Impfung mit mRNA-Impfstoff ab dem 2. Trimester ermöglicht wird [2, 19], wie bei (Eltern von) Kindern über 12 Jahren eine besondere Beratungssituation.

## Fazit für die Praxis

- Bei der Mehrzahl der Eltern besteht bei gleichzeitigem Vertrauen in nicht evidenzbasierte Behandlungsmethoden Unsicherheit bezüglich der Risiken und Nebenwirkungen einer COVID-19-Impfung.
- Mütter sind weniger bereit zu eigener Impfung und Kinderimpfung als Väter.
- In Übereinstimmung mit anderen Studien empfehlen wir eine faktenbasierte Impfberatung und eine Risikokommunikation mittels spezieller Grafiken, die das Verständnis numerischer Werte erhöhen und auf wissenschaftlich erprobten Kommunikationsformaten basieren.
